# Dual RNA-Seq Analysis of the Interaction Between Edible Fungus *Morchella sextelata* and Its Pathogenic Fungus *Paecilomyces penicillatus* Uncovers the Candidate Defense and Pathogenic Factors

**DOI:** 10.3389/fmicb.2021.760444

**Published:** 2021-12-02

**Authors:** Yang Yu, Hao Tan, Tianhai Liu, Lixu Liu, Jie Tang, Weihong Peng

**Affiliations:** ^1^National-Local Joint Engineering Laboratory of Breeding and Cultivation of Edible and Medicinal Fungi, Institute of Agricultural Resources and Environment, Sichuan Academy of Agricultural Sciences, Chengdu, China; ^2^National Observing and Experimental Station of Agricultural Microbiology, Ministry of Agriculture and Rural Affairs, Chengdu, China; ^3^School of Bioengineering, Jiangnan University, Wuxi, China

**Keywords:** *Morchella sextelata*, *Paecilomyces penicillatus*, pathogenic factors, response, transcriptomics, CAZymes, laccase, tyrosinase

## Abstract

Morels (*Morchella* spp.) are economically important mushrooms cultivated in many countries. However, their production and quality are hindered by white mold disease because of *Paecilomyces penicillatus* infection. In this study, we aimed to understand the genetic mechanisms of interactions between *P. penicillatus* and *Morchella*. *M. sextelata*, the most prevalent species of *Morchella* in China, was inoculated with *P. penicillatus*; then, the expression profiles of both fungi were determined simultaneously at 3 and 6 days post-inoculation (dpi) using a dual RNA-Seq approach. A total of 460 and 313 differentially expressed genes (DEGs) were identified in *P. penicillatus* and *M. sextelata*, respectively. The CAZymes of β-glucanases and mannanases, as well as subtilase family, were upregulated in *P. penicillatus*, which might be involved in the degradation of *M. sextelata* cell walls. Chitin recognition protein, caffeine-induced death protein, and putative apoptosis-inducing protein were upregulated, while cyclin was downregulated in infected *M. sextelata*. This indicates that *P. penicillatus* could trigger programmed cell death in *M. sextelata* after infection. Laccase-2, tyrosinases, and cytochrome P450s were also upregulated in *M. sextelata*. The increased expression levels of these genes suggest that *M. sextelata* could detoxify the *P. penicillatus* toxins and also form a melanin barrier against *P. penicillatus* invasion. The potential pathogenic mechanisms of *P. penicillatus* on *M. sextelata* and the defense mechanisms of *M. sextelata* against *P. penicillatus* were well described.

## Introduction

Morels (*Morchella* spp.), members of the Ascomycetes, Pezizomycetes, Pezizales, Morchellaceae, and *Morchella*, are commercially important edible mushrooms that are consumed throughout the world (Du et al., 2012). After more than 100years of trials, the commercial cultivation of *Morchella* in the field was successful (Ower et al., 1986; Dahlstrom et al., 2000; Masaphy, 2010). Since 2012, the industry that cultivates *Morchella* in China, with Sichuan as a typical example, developed rapidly (Peng et al., 2016). The cultivation area of morels in China has expanded from 200ha in 2012 to 10,000ha in 2020 (Zhao, 2021). The considerable economic benefits of morel cultivation have attracted many farmers and have become their main source of income. The *Morchella* species currently cultivated in China include *M. importuna*, *M. sextelata*, and *M. septimelata* (Liu et al., 2018). Among them, *M. sextelata* comprises >90% of the total area of *Morchella* cultivation (Deng et al., 2021). However, the white mold disease (WMD) caused by *Paecilomyces penicillatus* (Ascomycetes) seriously harms *Morchella* (He et al., 2017). WMD outbreak spreads quickly, infecting a large area. In severe cases, 60–80% of *Morchella* are infected in China, resulting in a decline in their production and commodity value (Chen et al., 2017; He et al., 2017). In addition, a high proportion of P. penicillatus in soil microbial community could inhibit the formation of f Morchella fruiting bodies (Tan et al., 2021a). A large number of farmers suffer financial hardship owing to WMD. Therefore, it is extremely urgent to prevent and control WMD.

Edible mushrooms are primarily cultivated and managed indoors. Therefore, the prevention and control of edible mushroom diseases rely on environmental controls during cultivation, including the control of temperature, light, water, and air, as well as the use of physical and chemical sterilization methods (Gea et al., 2021). *Morchella* is artificially cultivated in the field (Liu et al., 2018). Hence, the common strategies to prevent and control diseases of edible mushrooms are not effective for the control of *Morchella* diseases, such as WMD. Similar to other major crops, breeding resistant varieties are an effective way to control mushroom diseases (Kage et al., 2016). Several studies have addressed the immune response of mushrooms to mycoparasites, although this area of research is in its infancy. The activity of laccase, particularly encoded by *lcc2* gene, was found to contribute to the metabolism of toxin production in *Trichoderma aggressivum* and enhanced the resistance to green mold disease in *Agaricus bisporus* (Sjaarda et al., 2015). A total of 17 simple sequence repeat markers associated with resistance to *Mycogone perniciosa* in *A. bisporus* have been established (Fu et al., 2016). A quantitative trait loci analysis was used to identify the locations, numbers, and effects of genomic regions associated with resistance to *Lecanicillium fungicola* in *A. bisporus*, and four traits related to resistance were analyzed (Foulongne-Oriol et al., 2012). Further research and data mining will help design classical breeding and genetic modification schemes, thus, producing resistant varieties.

Recently, the genome-wide profiles of several species of *Morchella*, including *M. importuna, M. sextelata*, and *M*. *septimelata*, as well as that of the pathogen *P. penicillatus*, were reported (Wingfield et al., 2018; Mei et al., 2019; Tan et al., 2019; Wang et al., 2020). *P. penicillatus* was found to harbor many CAZymes, particularly clusters of chitinase genes, which could be related to its pathogenicity (Wang et al., 2020). Cheng et al. (2021) reported the regulatory mechanism of interaction between *P. penicillatus* and *M. importuna*. However, the lack of a control group of *M. importuna* at the same developmental stage complicated the conclusions from that study. To date, the genetic and pathogenic mechanisms that underlie the interaction between *P. penicillatus* and *Morchella* remain unclear, which makes it difficult to efficiently breed resistant varieties and develop specific biological or chemical methods to control WMD in *Morchella*. In this study, *M. sextelata*, the most prevalent species of *Morchella* in China, was selected as the research object and artificially inoculated with *P. penicillatus* to develop WMD in the field. The transcriptional changes of *P. penicillatus* in different infection stages were analyzed. The differences in transcriptional changes were compared between different growth stages of infected and healthy *M. sextelata*. We aimed to profile the infection mechanism of *P. penicillatus*, the response mechanism of *M. sextelata* to *P. penicillatus* infection, and the candidate defense genes of *M. sextelata* that protect against *P. penicillatus*. This will provide theoretical support for the effective control of WMD and reduce the economic risk caused by WMD in the cultivation of *Morchella*.

## Materials and Methods

### Fungal Strains and Experimental Treatment

Cultivated strains of *M. sextelata* were purchased from Jindi Tianlingjian Biotechnology Co., Ltd., in Chengdu, Sichuan, China. *P. penicillatus* was isolated in our previous study (He et al., 2017) and was deposited in the Culture Collection Center of the Soil and Fertilizer Institute, Sichuan Academy of Agricultural Sciences, Chengdu, China. The strains of *M. sextelata* used in this study were cultivated on a farm in Xindu, Chengdu, China (30.8°N, 104.2E). The cultivation, growth, and management of morels were done according to standard practices (Tan et al., 2021b). *P. penicillatus* was cultured on potato dextrose agar medium for 5 d at 25°C before inoculation. When the fruiting bodies of *M. sextelata* reached 7cm high, they were inoculated with *P. penicillatus* mycelia. Needle tip-sized mycelia were picked up with sterilized toothpicks and inoculated on *Morchella* fruiting bodies. A total of 120 *M. sextelata* fruiting bodies were used for the field experiment. Half of them were inoculated with *P. penicillatus* as explained above, while the remaining half were not inoculated. According to the phenotypic changes of the lesion, samples were collected at 3 and 6 days post-inoculation (dpi). One cm^2^ fruiting body centered on the lesion was collected for each inoculated morel, while healthy tissue from the same site was collected from the non-inoculated morels. The samples were divided into four groups, including non-inoculated groups on 3 and 6 dpi and inoculated groups on 3 and 6 dpi. Each group contained three biological replicates, and groups of six fruiting bodies were pulled into one biological replicate. All the samples were stored at −80°C for subsequent transcriptome analysis.

### RNA Extraction and qRT-PCR

Total RNA was isolated and purified from healthy and inoculated morels using the TRIzol reagent (Invitrogen, Carlsbad, CA, United States) following the manufacturer’s instructions. The purity, concentration, and integrity of each sample were determined using a NanoDrop spectrophotometer (Thermo Fisher Scientific, Inc., Waltham, MA, United States), a Qubit RNA Kit (Life Technologies, Carlsbad, CA, United States), and a 2100 Bioanalyzer (Agilent Technologies, Santa Clara, CA, United States), respectively.

About 2μg of total RNA from each sample was reverse-transcribed into cDNA using a RevertAid First Strand cDNA Synthesis Kit (THERMO, United Kingdom). The profiles of expression of the genes expression profiles were determined using SYBR Green Premix (TaKaRa Code RR820A; TaKaRa, Shiga, Japan) on a Roche LightCycle480 system (Roche Applied Science, Rotkreuz, Switzerland). All the PCR reactions were conducted using 40cycles at 98°C for 10s, 60°C for 10s, and 72°C for 10s, in a 20μl reaction mixture that contained 10pmol of each primer and 2μl of cDNA as a template. All the reactions were performed in triplicate, and 5.8S rRNA was used as the internal control for normalization. Supplementary Table S1 lists the primers used for quantitative real-time reverse transcriptase–PCR (qRT-PCR).

### cDNA Library Construction and Sequencing

cDNA library preparation and sequencing were conducted by the Personal Biotechnology Co., Ltd., in Shanghai, China. All the libraries were sequenced using an Illumina Nova-Seq platform (Illumina, San Diego, CA, United States). The FASTX toolkit was used to filter the raw data, short-fragment reads, and sequencing adapters, while other low-quality reads were filtered to obtain the clean reads. After preprocessing the RNA-Seq data, the reads were mapped to the *P. penicillatus* and *M. sextelata* reference genomes using HISAT2 software.^1^ The RNA reads were classified as *M. sextelata* or *P. penicillatus* based on their similarity to the corresponding genome sequence. Fragments per kilobase of transcript per million mapped fragments were used to normalize the abundance of transcripts (Trapnell et al., 2012).

### Differential Expression Analysis

Differential expression analysis was conducted using DESeq2 (Love et al., 2014). The resulting value of *p* were adjusted using Benjamini and Hochberg’s approach to control the false discovery rate. Genes with an adjusted *p* <0.05 and log2 (fold change) >1 that were detected by DESeq were designated as differentially expressed.

TopGO was used for the Gene Ontology (GO) enrichment analysis, and the calculated value of *p* was then obtained using the Wallenius non-central hypergeometric distribution method to identify significantly enriched GO terms (*p* <0.05) from all of the DEGs (Ashburner et al., 2000). Kyoto Encyclopedia of Genes and Genomes (KEGG) pathway enrichment analyses were performed on the DEGs using clusterProfiler. The value of *p* was calculated using the hypergeometric distribution method (Kanehisa et al., 2004).

### Data Availability

The Illumina Nova-Seq sequencing data from this study were deposited in the NCBI Sequence Read Archive.^2^ The reference genome of *P. penicillatus* can be downloaded from https://www.ncbi.nlm.nih.gov/genome/79861?genome_assembly_id=1473685, and the reference genome of *M. sextelata* can be downloaded from https://www.ncbi.nlm.nih.gov/genome/86229?genome_assembly_id=748597.

## Results

### Overview of the RNA-Seq Data

**Figure 1:**
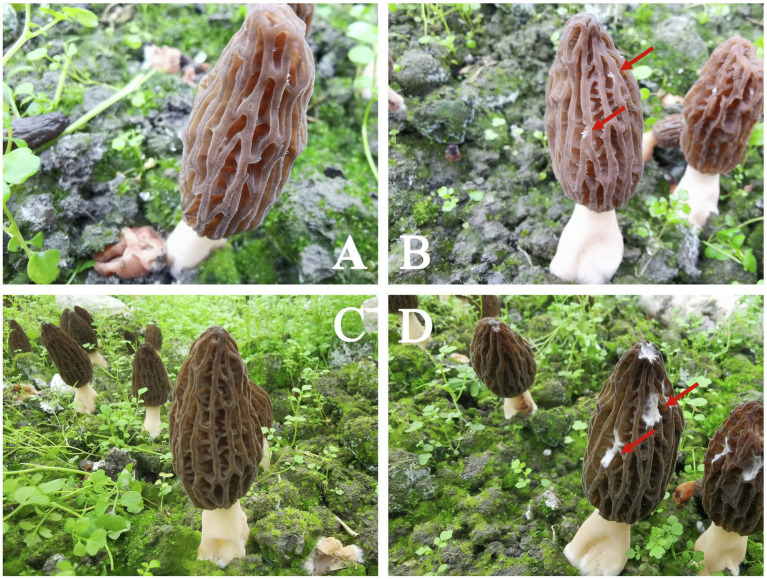
Field phenotype of the white mold disease (WMD) in *Morchella sextelata*. **(A,C)** represent the non-inoculated morels at 3 and 6days, respectively. **(B,D)** represent the morels at 3 and 6days post-*Paecilomyces penicillatus* inoculation, respectively. The red arrow shows the WMD symptom.

White spots were observed on the *M. sextelata* fruiting bodies at 3days after inoculation with *P. penicillatus*, indicating that *P. penicillatus* was successfully germinated and began to infect *Morchella*. The typical symptom of white mold disease started to appear at 6 dpi (Figure 1). Based on the observed phenotypic change, we sampled tissues at 3 and 6 dpi, respectively. The non-inoculated tissues at 3 and 6 dpi were used as the control groups for the subsequent dual RNA-Seq analysis.

An average of 75,728,447 raw reads was generated for each sample with a Q20 and Q30 of 97.81 and 94.11%, respectively (Supplementary Table S2). After quality control, an average of 68,239,578 clean reads was generated. All the clean reads were mapped to a corresponding reference genome. An average of 92.87% clean reads of the non-inoculated samples was mapped to the *M. sextelata* genome, while none (< 0.05%) was mapped to the *P. penicillatus* genome. A total of 90.56 and 84.72% clean reads of the inoculated samples were mapped to the genome of *M. sextelata* at the early and late stages of infection, respectively. However, 2.01 and 6.85% clean reads from the infected sites were mapped to the *P. penicillatus* genome at the early and late stages of infection, respectively (Supplementary Table S3).

### Quantitative RT-PCR Validation

**Figure 2:**
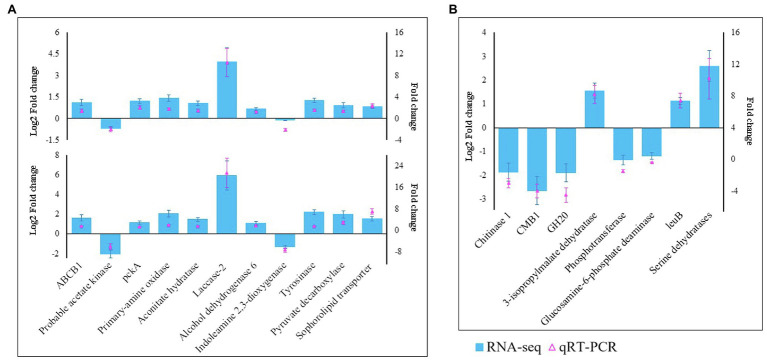
Validation of the RNA-Seq data by qRT-PCR. **(A)** 11 DEGs from *M. sextelata* were selected for validation, and the y-axis shows the fold change expression at 3 and 6 days post-inoculation (dpi) compared with the non-inoculated samples. **(B)** Eight DEGs from *P. penicillatus* were selected, and the y-axis shows the fold change expression at 6 dpi compared with 3 dpi. Each data point was obtained from three biological replicates. DEGs, differentially expressed genes; qRT-PCR, quantitative real-time reverse transcriptase–PCR.

We selected 19 genes, including 11 (laccase-2, ABCB1, tyrosinase, primary amine oxidase, pckA, probable acetate kinase, aconitate hydratase, alcohol dehydrogenase 6, indoleamine 2,3-dioxygenase, pyruvate decarboxylase, and sophorolipid transporter) from *M. sextelata* and eight (chitinase 1, CMB1, GH20, 3-isopropylmalate dehydratase, phosphotransferase, glucosamine-6-phosphate deaminase, leuB, and serine dehydratases) from *P. penicillatus*, to validate the RNA-Seq data. qRT-PCR assays were conducted to test their patterns of expression at 3 and 6 dpi. As shown in Figure 2, the qPCR assay identified nine upregulated genes in *M. sextelata* and two were downregulated. Among them, laccase-2 was highly upregulated at both infection stages by 10.9- (3 dpi) and 21.4-fold (6 dpi). In *P. penicillatus*, three were upregulated, and two were downregulated. The patterns of expression obtained from the qRT-PCR were consistent with the RNA-Seq results, indicating that the RNA-Seq data were well suited to analyze the interaction between *P. penicillatus* and *M. sextelata*.

### Differentially Expressed Genes in *P. penicillatus*

**Figure 3:**
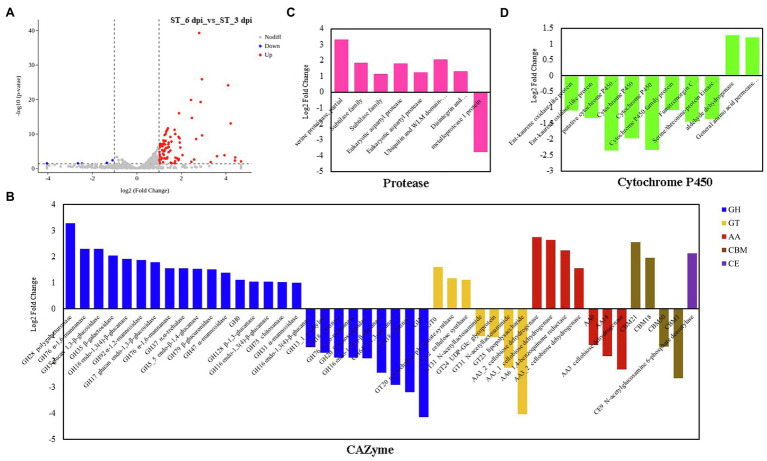
DEGs at 6 days post-inoculation (dpi) compared with 3 dpi in *Paecilomyces penicillatus*. **(A)** Volcano plot of the DEGs in *P. penicillatus*. Differentially expressed CAZymes **(B)**, proteases **(C)** and cytochrome P450s **(D)** in *P. penicillatus* during the infection process. ST, inoculated samples; 3 and 6 dpi, 3 and 6 days post-inoculation; DEGs, differentially expressed genes.

The transcriptional expression of *P. penicillatus* on 3 and 6 dpi was analyzed. A total of 460 DEGs in *P. penicillatus* were identified by comparing the abundance of transcripts on 6 dpi with those on 3 dpi. A total of 336 were upregulated, and 124 were downregulated. Two proteases, including subtilase family protease and serine proteinase, were the most highly expressed among these DEGs (Figure 3A; Supplementary Table S4). The expressions of CAZymes in *P. penicillatus*, which play an important role in mycoparasitic fungi (Wang et al., 2020), were analyzed. A total of 45 CAZymes were identified from all the DEGs in *P. penicillatus* (Figure 3B), including 25 glycosyl hydrolases (GHs), seven glycosyltransferases (GTs), seven auxiliary activities (AAs) enzymes, four carbohydrate-binding modules, and one carbohydrate esterase (CE). Among them, GH 17 (glucan endo-1,3-β-glucosidase), AA6 (1,4-benzoquinone reductase), and GT2 (chitin synthase 1) were the top three genes that were expressed, and they were all upregulated during the late stage of *P. penicillatus* infection (Supplementary Table S4), indicating their importance in the pathogenicity of *P. penicillatus*. Out of the 25 GHs, six β-glucanases, three α-mannosidases, and two α-1,6-mannanases were upregulated, while two chitinases (GH18) were downregulated (Figure 3B). This information indicated that the main cell wall-degrading enzymes (CWDEs) were glucanases and mannanases rather than chitinase when *P. penicillatus* infected *M. sextelata*.

### Functional Analysis of Differentially Expressed Genes in *P. penicillatus*

**Figure 4:**
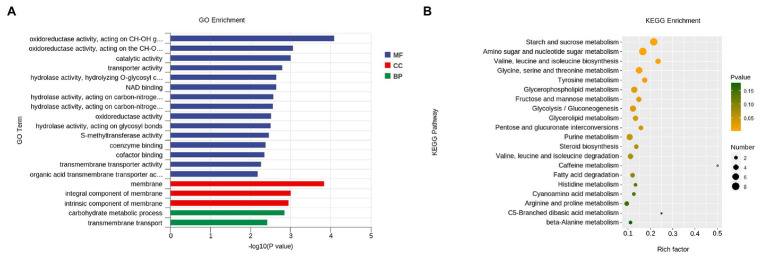
Functional enrichment analyses for DEGs in *P. penicillatus* during the infection process. **(A)** GO enrichment analyses. GO term enrichment values of *p* are indicated on the x-axis. BP, biological process; MF, molecular function; CC, cellular component. **(B)** KEGG enrichment analyses. The x-axis represents the rich factor. The size of the dot indicates the number of DEGs involved in the pathway. Color bars on the right represent the value of *p* of the KEGG pathway. DEGs, differentially expressed genes; GO, gene ontology; KEGG, kyoto encyclopedia of genes and genomes.

GO category enrichment analysis was utilized to elucidate the functional enrichment of the DEGs in *P. penicillatus*. A total of 73 GO terms were enriched (*p*<0.05) in *P. penicillatus* (Supplementary Table S5). Four “hydrolase activity (GO:0004553, GO:0016813, GO:0016810, and GO:0016798),” three “oxidoreductase activity (GO:0016614, GO:0016616, and GO:0016491),” one “NAD binding (GO:0008172)” in the category of molecular function, one “carbohydrate metabolic process (GO:0005975)” in the biological processes category, and three “membrane (GO:0016020, GO:0016021, and GO:0031224)” in the cellular component category were among the top 20 GO terms (Figure 4). The DEGs in the “carbohydrate metabolic process” were analyzed. Among the 34 DEGs, 16 of them were CAZymes and also belonged to the GO term of “hydrolase activity, hydrolyzing O-glycosyl compounds” (Supplementary Table S5). Eight proteases were identified from “hydrolase activity,” including three metalloprotease proteins, two proteinase T-like proteins, two eukaryotic aspartyl proteases, and one serine proteinase (Figure 3C), which could act as CWDEs when *P. penicillatus* infected the morels. Ten cytochrome P450 (CYP) superfamily proteins were identified in the GO term of oxidoreductase activity, and eight of them were downregulated (Figure 3D).

The KEGG pathway analysis showed that 11 pathways were significantly enriched (*p*<0.05) in *P. penicillatus*, including five “carbohydrate metabolism,” three “amino acid metabolism,” two “lipid metabolism,” and one “nucleotide metabolism.” Among them, “starch and sucrose metabolism (ko00500)” and “amino sugar and nucleotide sugar metabolism (ko00520)” were the two most significantly enriched pathways (Figure 4; Supplementary Table S5). These results indicate that carbohydrate metabolism plays an important role in the pathogenicity of *P. penicillatus* on *M. sextelata*, which was similar to the results of GO category enrichment analysis.

### Differentially Expressed Genes in *M. sextelata*

**Figure 5:**
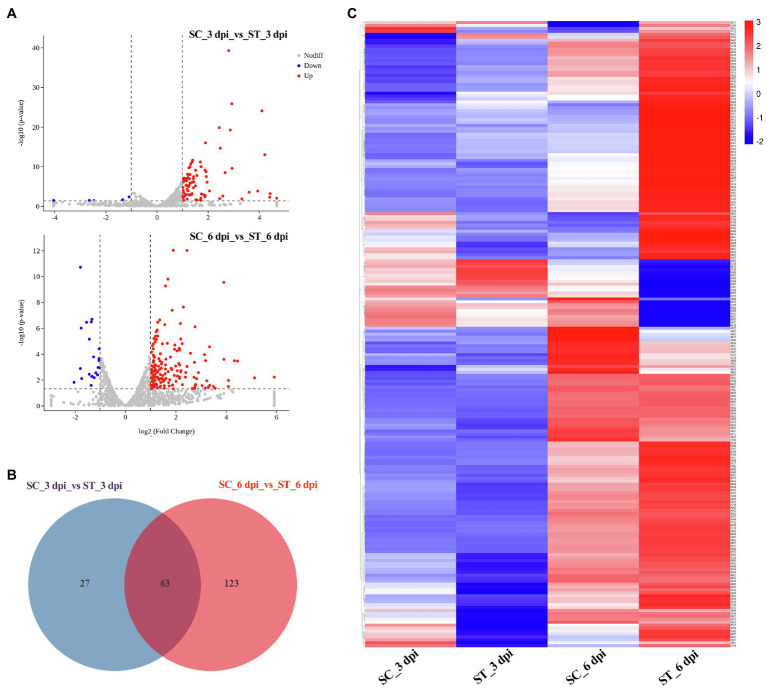
DEGs in *M. sextelata* at 3 and 6 days post-inoculation. Volcano plot **(A)**, Venn diagrams **(B)** and heat maps **(C)** of the DEGs modulated by *P. penicillatus* infection in *M. sextelata*. Blue indicates downregulated DEGs, while red indicates upregulated DEGs in heat maps. ST and SC represent the inoculated and non-inoculated samples, respectively. DEGs, differentially expressed genes; dpi, days post-inoculation.

We also analyzed the transcriptional expression of *M. sextelata* after infection with *P. penicillatus*. A total of 313 DEGs were identified in *M. sextelata* by comparing the abundance of transcripts of the inoculated morels with those of the non-inoculated morels, and 90 and 186 DEGs were identified on 3 and 6 dpi, respectively. Among them, 63 genes were common DEGs in the two infection stages (Figure 5; Supplementary Table S6). Notably, all the 63 common DEGs were upregulated during the two infection stages. Among them, a laccase-2 gene was the most highly upregulated (log2fc=5.9; Supplementary Table S6). Interestingly, chitin recognition protein, cyclin-dependent protein kinase, caffeine-induced death protein 2, and allergen Asp f 15 precursor involved in immune responses were identified in these common DEGs (Supplementary Table S6). A total of 85 upregulated and five downregulated genes were found to be the specific DEGs on 3 dpi in *M. sextelata*, while 164 upregulated and 22 downregulated genes were specifically identified on 6 dpi. A cyclin was downregulated on 3 dpi and expressed at normal levels on 6 dpi (Supplementary Table S6).

### Functional Analysis of Differentially Expressed Genes in *M. sextelata*

**Figure 6:**
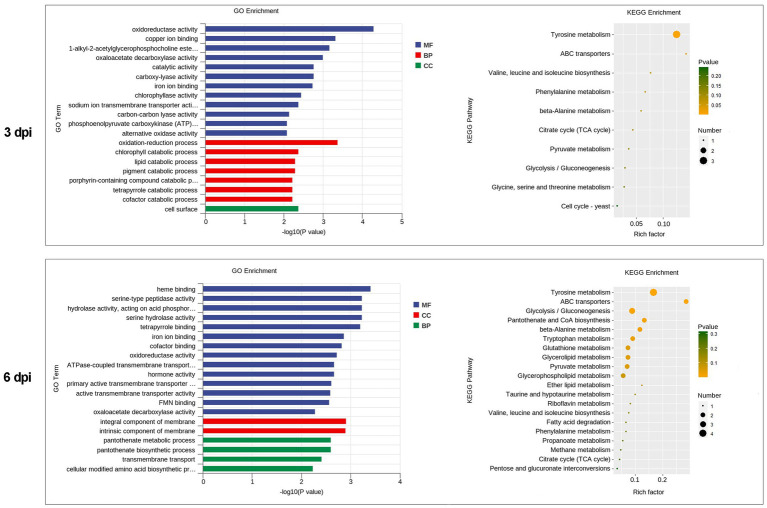
Functional enrichment analyses for DEGs in *M. sextelata* at 3 and 6 days post-inoculation. GO enrichment analyses, GO term enrichment values of *p* are indicated on the x-axis. BP, biological process; MF, molecular function; CC, cellular component. KEGG enrichment analyses, x-axis represents the rich factor; the dot size indicates the number of DEGs involved in the pathway; Color bars on the right represent the values of *p* of the KEGG pathway. 3 and 6 dpi represent 3 and 6 days post-inoculation. DEGs, differentially expressed genes; GO, gene ontology; KEGG, kyoto encyclopedia of genes and genomes.

**Figure 7:**
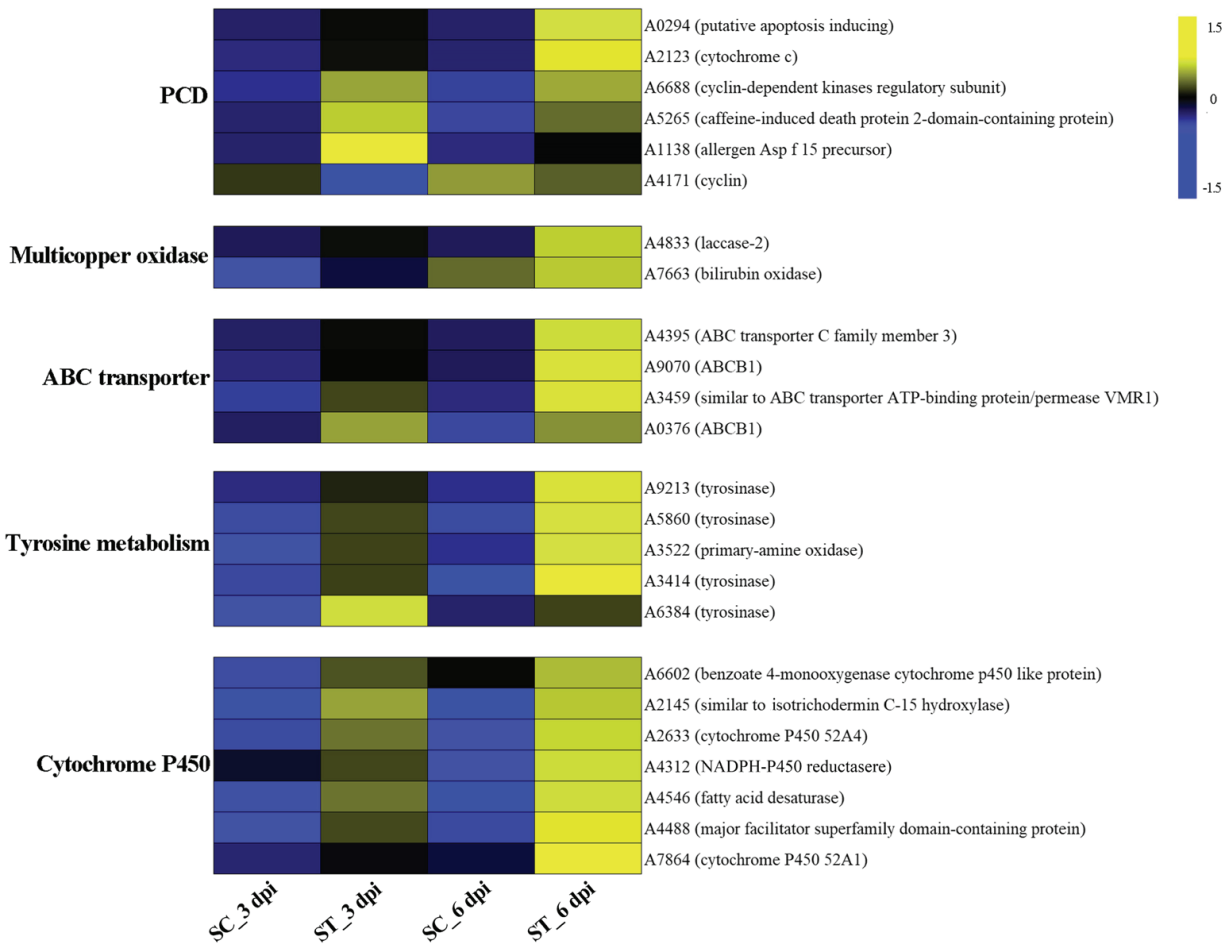
Heat map of DEGs that responded to *P. penicillatus* infection in *M. sextelata*. PCD, programmed cell death; ST and SC represent the inoculated and non-inoculated samples, respectively; dpi, days post-inoculation; blue indicates downregulated DEGs, while yellow indicates upregulated DEGs. KEGG, kyoto encyclopedia of genes and genomes.

GO category enrichment analysis was conducted in more detail, and 77 and 71 GO terms were identified (*p*<0.05) on 3 and 6 dpi, respectively (Supplementary Table S7). “Oxidoreductase activity (GO:0016491),” “oxidation-–reduction process (GO:0055114),” “carboxy-lyase activity (GO:0016831),” and several pathways associated with these GO terms were commonly enriched in both infection stages (Figure 6; Supplementary Table S7). DEGs in the oxidation-reduction process were screened, and sets of genes associated with immunity were identified, including five CYPs (benzoate 4-monooxygenase CYP-like protein, NADPH-P450 reductase, CYP52A4, CYP52A4, and similar to isotrichodermin C-15 hydroxylase), four tyrosinases, three flavin oxidoreductases, two multicopper oxidases (laccase-2 and bilirubin oxidase), one copper amine oxidase, and one cytochrome c peroxidase (Figure 7; Supplementary Table S8), were all upregulated after infection with *P. penicillatus* and could be related to the detoxification of *P. penicillatus* toxins. In addition, a putative apoptosis-inducing protein was upregulated (Figure 7; Supplementary Table S8). GO terms as serine-type peptidase activity (GO:0008236), hormone activity (GO:0005179), pantothenate metabolic process (GO:0015939), and FMN binding (GO:0010181) were specifically enriched on 6 dpi (Supplementary Table S7), indicating that *M. sextelata* could distinguish between the early and late stages of infection with *P. penicillatus*.

KEGG pathway analysis identified two and nine significantly enriched pathways on 3 and 6 dpi, respectively. Among them, “tyrosine metabolism (ko00350)” and “ABC transporters (ko02010)” were the common enriched pathways in both infection stages (Supplementary Table S7). The DEGs in these two pathways were screened. Five tyrosinases and two ABC transporter family proteins were found to be upregulated in the infected morel, confirming their important roles in the response of *M. sextelata* to *P. penicillatus* (Figure 7; Supplementary Table S8). “Glycolysis/Gluconeogenesis (ko00010),” “pantothenate and CoA biosynthesis (ko00770),” and several other pathways were specifically enriched on 6 dpi (Supplementary Table S7), which is similar to the results of GO category enrichment analysis.

## Discussion

Owing to the lack of resistant varieties and the prevalence of *P. penicillatus* in many regions, WMD poses a serious threat to the cultivation of morels in China. This study identified a number of genes involved in the regulation of the pathogenicity of *P. penicillatus* to *M. sextelata* and the response of *M. sextelata* to infection with *P. penicillatus*. We defined how *P. penicillatus* successfully parasitizes *M. sextelata* and how *M. sextelata* encodes its immune response to invasion by *P. penicillatus*.

Mycoparasitic fungi usually produce various fungal CWDEs to lyse the host cell wall, including glucanases, chitinase, and proteases (Gruber and Seidl-Seiboth, 2012). A previous study found that *P. penicillatus* encodes approximately 300 CAZymes, which could be involved in the degradation of fungal cell walls (Wang et al., 2020). *P. penicillatus* activated six β-glucanases, three α-mannosidases, and two α-1,6-mannanases when it infected *M. sextelata* (Figure 3B), indicating that glucanases and mannanases were the primary CAZymes of *P. penicillatus* that are involved in the degradation of *M. sextelata* cell walls. Only two chitinases (GH18) were identified in *P. penicillatus*, even though they were the most annotated CAZyme family in the *P. penicillatus* genome (Wang et al., 2020). They were all unexpectedly downregulated (Figure 3B). Previous studies have shown that mycoparasitic species of *Trichoderma* activated chitinase when interacting with *A. bisporus* (Guthrie and Castle, 2006; Ihrmark et al., 2010), in contrast to the findings of this study. However, the chitinases of *T. gamsii* were all downregulated when interacting with *Fusarium graminearum* (Zapparata et al., 2021), which was consistent with the data of this study. This suggests that *P. penicillatus* could change the expression of chitinases based on demands of specific situations when infecting other Ascomycetes fungi. In this study, chitin synthase 1 was upregulated in *P. penicillatus*. One possible explanation for this upregulation is that these chitinases were involved in the cell wall remodeling during *P. penicillatus* growth. Their downregulation could result in strengthened cell walls during interaction. Another possible explanation is that when in contact with *M. sextelata*, *P. penicillatus* suppresses its secretion of fungal chitinases to avoid recognition and the induction of defense reactions in *M. sextelata*. Several proteases, including subtilase family protease, metalloprotease, and serine proteinase, were activated during the infection process of *P. penicillatus* (Figure 3C). These genes were reported to be involved in the regulation of cell wall degradation in plants and microorganisms (Feng et al., 2014; Schaller et al., 2018). In particular, the subtilase family proteases are good candidates for this function since they were reported to be induced during mycoparasitism before and during contact with the host in different *Trichoderma* species (Suárez et al., 2007; Seidl et al., 2009). This indicated that these CWDEs play an important role in the parasitism of *P. penicillatus* on morels.

*M. sextelata* activated a series of mechanisms to respond once it sensed the invasion of *P. penicillatus*. A chitin recognition protein and an allergen Asp f 15 precursor in *M. sextelata* were upregulated during both infection stages (Figure 7; Supplementary Table S8), which could activate the downstream immune response (Sanchez-Vallet et al., 2015). *M. sextelata* upregulated a caffeine-induced death protein 2-domain-containing protein and a putative apoptosis-inducing protein, as well as downregulating a cyclin (Figure 7; Supplementary Table S8); these genes are widely reported to promote immunity by initiating programmed cell death (Saiki et al., 2011; Qi and Zhang, 2019). We hypothesize that *M. sextelata* activates an apoptotic response to escape the proliferation of the pathogen when *P. penicillatus* invades.

A change in the reduction-oxidation status is one of the earliest responses detected when cells are attacked (Frederickson Matika and Loake, 2014). The reduction-oxidation process plays a key role in both the host and pathogen during the interaction of *P. penicillatus* and *M. sextelata*, while the regulatory mechanisms differ. The cytochrome P450 family genes in *M. sextelata* and *P. penicillatus* showed an opposite trend of expression. Six of the seven cytochrome P450 in *P. penicillatus* were downregulated, while all five in *M. sextelata* were upregulated (Figures 3D, 7). A previous study reported that ent-kaurene-derived diterpenoids act as a virulence factor interacting with the bacterium *Xanthomonas oryzae* pv*. oryzicola* and rice (Lu et al., 2015) and inhibited the growth of *Staphylococcus aureus* and *Bacillus subtilis* (Fatope et al., 2010). Two ent-kaurene oxidase-like proteins that are cytochrome P450s were downregulated in *P. penicillatus*, which could lead to increased synthesis of diterpenoid cytotoxins in *P. penicillatus* (Wang et al., 2012). Once the diterpenoid cytotoxins were secreted into *M. sextelata* cells, the pathogenicity of *P. penicillatus* to *M. sextelata* might be enhanced. Interestingly, one isotrichodermin C-15 hydroxylase gene was downregulated in *P. penicillatus*, while another was upregulated in *M. sextelata*. Previous study reported that blocking an isotrichodermin C-15 hydroxylase in *Fusarium sporotrichioides* resulted in the accumulation of trichothecenes, a virulence factor of *Fusarium*. spp. (McCormick and Hohn, 1997; Ward et al., 2002). This study also observed increased expression of CYPs superfamily genes in *M. sextelata* after infection with *P. penicillatus*, including benzoate 4-monooxygenase CYP-like protein and NADPH-P450 reductase. Benzoate 4-monooxygenase of ascomycete *Cochliobolus lunatus* was reported to be involved in detoxification of benzoic acid (BA), a key intermediate in metabolism of aromatic compounds in fungi, as well as the detoxification of phenolic compounds (Podobnik et al., 2008; Berne et al., 2012). NADPH-P450 reductase was reported to plays a central role in chemical detoxification and insecticide resistance in insects and fungi (Lian et al., 2011; Xu et al., 2021). This information suggests that these CYPs in *M. sextelata* might participated in the detoxification of toxic metabolites produced by *P. penicillatus*. We also identified two ABCB1 proteins that were upregulated in *M. sextelata* following infection with *P. penicillatus* (Figure 7). We hypothesize that *M. sextelata* could transport harmful substances secreted by *P. penicillatus* out of its cells by activating the expression of ABCB1. This has been confirmed by previous studies that ABCB1, a member of the ABC transporter family, plays an important physiological role in protecting the tissues from xenobiotics and endogenous metabolites (Roy et al., 2021).

In fungi, tyrosinases are generally associated with the formation and stability of spores, defense and virulence, and melanin production (Halaouli et al., 2006). Once the cells have been damaged, melanin produced by the reaction of tyrosinase with its substrate forms a melanin barrier to protect against the invasion of pathogenic bacteria or their toxins (Janusz et al., 2020). In this study, tyrosinase metabolism was the most enriched KEGG pathway in the infected *M. sextelata* (Figure 6). Five tyrosinase genes in *M. sextelata* were upregulated after infection by *P. penicillatus* (Figure 7). This indicated their importance in *M. sextelata* against infection with *P. penicillatus*.

Fungal laccase, a multicopper oxidase, is usually induced by a variety of phenolic compounds (Piscitelli et al., 2011) and was reported to have a wide range of functions, including defense against stressful conditions (Lakshmanan and Sadasivan, 2016; Chakraborty et al., 2020). One 1,4-benzoquinone reductase was upregulated in *P. penicillatus* (Figure 3B; Supplementary Table S4) and could be involved in the downstream synthesis of phenolic compounds. Interestingly, laccase-2 was the most upregulated gene in infected *M. sextelata* (Figure 7; Supplementary Table S6). A previous study found that the activity of laccase-2 enhanced the resistance of *A. bisporus* to the *T. aggressivum* toxin (Sjaarda et al., 2015), which is 3,4-dihydro-8-hydroxy-3-methyl isocoumarin that contains a phenolic hydroxyl and lactone ring (Krupke et al., 2003). This suggests that *P. penicillatus* could synthesize phenolic toxins and secrete them into *M. sextelata*, and *M. sextelata* could detoxify these phenolic toxins by activating laccase expression. However, further experiments are needed to verify this hypothesis.

## Conclusion

During *P. penicillatus* infection of *M. sextelata*, cell wall-degrading enzymes, such as glucanases, mannanases, and proteases were probably secreted by *P. penicillatus* to degrade the cell wall of *M. sextelata*. *M. sextelata* triggered programmed cell death to prevent the excessive proliferation of *P. penicillatus* and synthesize cytochrome P450 and laccase to detoxify the *P. penicillatus* toxins. The melanin barrier formed by tyrosinase was also a possible immune pathway in *M. sextelata*. Although these possible regulatory pathways proposed in this study still need further verification, they provided a theoretical basis for researchers to breed WMD-resistant varieties and develop prevention and control methods for WMD.

## Data Availability Statement

The datasets presented in this study can be found in online repositories. The names of the repository/repositories and accession number(s) can be found in the article/Supplementary Material.

## Author Contributions

YY: conceptualization, funding acquisition, validation, investigation, data curation, visualization, writing – original draft preparation, and writing – review and editing. HT: funding acquisition, validation, and investigation. TL: investigation and data curation. LL: resources and investigation. JT: resources. WP: funding acquisition and supervision. All authors contributed to manuscript revision, read, and approved the submitted version.

## Funding

This research was funded by National Science Foundation of China (NSFC31901119), Science and Technology Project of Sichuan Province (2021YFYZ0026), SAAS-International Cooperation Project (2021ZSSFGH04), Edible Fungus Innovation Team of Sichuan Province (SCCXTD-2021-7), and Special Fund for Talent Introduction and Training of SAAS (510000-01-114852).

## Conflict of Interest

The authors declare that the research was conducted in the absence of any commercial or financial relationships that could be construed as a potential conflict of interest.

## Publisher’s Note

All claims expressed in this article are solely those of the authors and do not necessarily represent those of their affiliated organizations, or those of the publisher, the editors and the reviewers. Any product that may be evaluated in this article, or claim that may be made by its manufacturer, is not guaranteed or endorsed by the publisher.

## Footnotes

^1^ http://ccb.jhu.edu/software/hisat2/index.shtml

^2^ https://www.ncbi.nlm.nih.gov/sra/
