# Supplementary material for: Dual RNA-Seq Analysis of the Interaction Between Edible Fungus Morchella sextelata and Its Pathogenic Fungus Paecilomyces penicillatus Uncovers the Candidate Defense and Pathogenic Factors
Source: Front Microbiol. 2021 Dec 2;12:760444. doi: 10.3389/fmicb.2021.760444 (PMC8675245; doi:10.3389/fmicb.2021.760444)
Supplement: Supplementary file 1 [file Data_Sheet_1.ZIP › supplementary/Table S1.doc]

**Table S1** The primer for qRT-PCR used in this study.

|  | Gene_id | Annotation | Name of the primer | Sequence (5' - 3') |
| --- | --- | --- | --- | --- |
| *Morchella sextelata* | A0376 | ABCB1 | A0376-F | ATTGGCGGAGGAGGTATT |
|  |  |  | A0376-R | CTGTTGCTTGAGGTGCTATG |
|  | A1328 | Probable acetate kinase | A1328-F | GTGACCGAAGTAGCGGAA |
|  |  |  | A1328-R | GTGGAAGACCAGTGATGGA |
|  | A1746 | pckA | A1746-F | GGTGAGATGAAGAAGGGTGT |
|  |  |  | A1746-R | TTGGCAGAAGAGTGGAGAG |
|  | A3522 | Primary-amine oxidase | A3522-F | CGATGGAAACTGGACACAC |
|  |  |  | A3522-R | CGACACTGAATCTTTGACCTT |
|  | A4012 | Aconitate hydratase | A4012-F | GCACAAACTCCCGTATTGA |
|  |  |  | A4012-R | TCCCAAGCACATAGAGCA |
|  | A4833 | Laccase-2 | A4833-F | CAGGACGGTGTGAATGGT |
|  |  |  | A4833-R | TGGAGGGAGAAGTGAGAGTG |
|  | A4925 | Alcohol dehydrogenase 6 | A4925-F | GGTATCGGTGGTCTTGGA |
|  |  |  | A4925-R | GGTATCGGTGGTCTTGGA |
|  | A5030 | Indoleamine 2,3-dioxygenase | A5030-F | ATCCTCAATCTGCGGTCTT |
|  |  |  | A5030-R | GGGTCTGTCTTTCAACAATCTC |
|  | A5860 | Tyrosinase | A5860-F | AACTTTCTCGGCATCGTC |
|  |  |  | A5860-R | CACATCAAAACTCGGCTCT |
|  | A5869 | Pyruvate decarboxylase | A5869-F | CTTCTTCTTCACCACACCCT |
|  |  |  | A5869-R | TGTCTCTTCGTTGTTCGGT |
|  | A9070 | Sophorolipid transporter | A9070-F | CCTCGCAACCTTCTTCAC |
|  |  |  | A9070-R | CTAAATGCCGTCGTATTCCT |
| *Paecilomyces penicillatus* | A1236 | Chitinase 1 | A1236-F | GACGCTCTTTCCAACAAACT |
|  |  |  | A1236-R | GTAGTCCCAGATACCATTCTCC |
|  | A1964 | CMB1 | A1964-F | GAAGCCACCACGAACAAC |
|  |  |  | A1964-R | GACAAAGTCAAAGGGAACCTC |
|  | A2000 | GH20 | A2000-F | AGCCAACAACTCCATCCTC |
|  |  |  | A2000-R | CACTCCAAGACCATCTCCTC |
|  | A2102 | 3-isopropylmalate dehydratase | A2102-F | GAGAACCCCGACTTTGTG |
|  |  |  | A2102-R | TGTTGGTGATGGTGATGG |
|  | A3294 | Phosphotransferase | A3294-F | GACATCCCTCATCTCACATTC |
|  |  |  | A3294-R | TAATCAGTTGCTGGTCGCT |
|  | A3295 | Glucosamine-6-phosphate deaminase | A3295-F | TAATCAGTTGCTGGTCGCT |
|  |  |  | A3295-R | GAATAATAACAACCACCTCCCT |
|  | A6298 | leuB | A6298-F | ACTCTCTGAATCTCGGCAAC |
|  |  |  | A6298-R | CATCTCCTGCTTCTGTGGT |
|  | A7022 | Serine dehydratases | A7022-F | GCGAGAACTACCTGGAAAAG |
|  |  |  | A7022-R | CCATTGAACAGACCACCAC |
| Internal reference | 5.8 S | | 5.8 S-F | CTCTTGGCTCTCGCATCGAT |
|  |  |  | 5.8 S-R | GGCGCAATGTGCGTTCA |
