# Supplementary material for: Dual RNA-Seq Analysis of the Interaction Between Edible Fungus Morchella sextelata and Its Pathogenic Fungus Paecilomyces penicillatus Uncovers the Candidate Defense and Pathogenic Factors
Source: Front Microbiol. 2021 Dec 2;12:760444. doi: 10.3389/fmicb.2021.760444 (PMC8675245; doi:10.3389/fmicb.2021.760444)
Supplement: Supplementary file 1 [file Data_Sheet_1.ZIP › supplementary/Table S2.doc]

| Sample | Raw_Reads | Raw_Q20 (%) | Raw_Q30 (%) | Clean reads | Useful_read (%) |
| --- | --- | --- | --- | --- | --- |
| SC3d_1 | 84174150 | 97.9 | 94.37 | 75278074 | 89.43 |
| SC3d_2 | 68576512 | 97.84 | 94.15 | 62489802 | 91.12 |
| SC3d_3 | 76606844 | 97.66 | 93.74 | 69403978 | 90.59 |
| SC6d_1 | 73099352 | 97.91 | 94.3 | 66835764 | 91.43 |
| SC6d_2 | 71556912 | 97.7 | 93.8 | 64550772 | 90.2 |
| SC6d_3 | 74550470 | 97.88 | 94.31 | 66351034 | 89 |
| ST3d_1 | 75987052 | 97.79 | 94.05 | 68493054 | 90.13 |
| ST3d_2 | 75107316 | 97.9 | 94.28 | 67873364 | 90.36 |
| ST3d_3 | 72414372 | 97.7 | 93.84 | 65908174 | 91.01 |
| ST6d_1 | 80398738 | 97.78 | 94.08 | 72123024 | 89.7 |
| ST6d_2 | 76577130 | 97.88 | 94.32 | 68880822 | 89.94 |
| ST6d_3 | 79692518 | 97.77 | 94.11 | 70687076 | 88.69 |

**Table S2** Statistics of raw and clean data.

ST and SC represent the inoculated and non-inoculated samples, respectively. 3d, 3 days post inoculation (dpi); 6d, 6 dpi. Each group contained three biological replicates.
