# Supplementary material for: Dual RNA-Seq Analysis of the Interaction Between Edible Fungus Morchella sextelata and Its Pathogenic Fungus Paecilomyces penicillatus Uncovers the Candidate Defense and Pathogenic Factors
Source: Front Microbiol. 2021 Dec 2;12:760444. doi: 10.3389/fmicb.2021.760444 (PMC8675245; doi:10.3389/fmicb.2021.760444)
Supplement: Supplementary file 1 [file Data_Sheet_1.ZIP › supplementary/Table S3.doc]

**Table S3** The mapping efficiency for *Paecilomyces penicillatus* and *Morchella sextelata*.

| Sample | Clean_Reads | *Paecilomyces penicillatus* | | | *Morchella sextelata* | | |
| --- | --- | --- | --- | --- | --- | --- | --- |
|  |  | Total_Mapped | Multiple_Mapped | Uniquely_Mapped | Total_Mapped | Multiple_Mapped | Uniquely_Mapped |
| SC3d_1 | 75278074 | 42377 (0.06%) | 15293 (36.09%) | 27084 (63.91%) | 69596622 (92.45%) | 591380 (0.85%) | 69005242 (99.15%) |
| SC3d_2 | 62489802 | 31649 (0.05%) | 11343 (35.84%) | 20306 (64.16%) | 58093509 (92.96%) | 437660 (0.75%) | 57655849 (99.25%) |
| SC3d_3 | 69403978 | 32519 (0.05%) | 10153 (31.22%) | 22366 (68.78%) | 64536491 (92.99%) | 465772 (0.72%) | 64070719 (99.28%) |
| SC6d_1 | 66835764 | 30456 (0.05%) | 11069 (36.34%) | 19387 (63.66%) | 62515708 (93.54%) | 415106 (0.66%) | 62100602 (99.34%) |
| SC6d_2 | 64550772 | 30536 (0.05%) | 11473 (37.57%) | 19063 (62.43%) | 59637842 (92.39%) | 465297 (0.78%) | 59172545 (99.22%) |
| SC6d_3 | 66351034 | 29177 (0.04%) | 10627 (36.42%) | 18550 (63.58%) | 61616290 (92.86%) | 412099 (0.67%) | 61204191 (99.33%) |
| ST3d_1 | 68493054 | 938276 (1.37%) | 17624 (1.88%) | 920652 (98.12%) | 62324220 (90.99%) | 475094 (0.76%) | 61849126 (99.24%) |
| ST3d_2 | 67873364 | 1526345 (2.25%) | 57450 (3.76%) | 1468895 (96.24%) | 61193812 (90.16%) | 737128 (1.20%) | 60456684 (98.80%) |
| ST3d_3 | 65908174 | 1587684 (2.41%) | 32001 (2.02%) | 1555683 (97.98%) | 59662234 (90.52%) | 503340 (0.84%) | 59158894 (99.16%) |
| ST6d_1 | 72123024 | 3116684 (4.32%) | 37247 (1.20%) | 3079437 (98.80%) | 63385766 (87.89%) | 508788 (0.80%) | 62876978 (99.20%) |
| ST6d_2 | 68880822 | 4536429 (6.59%) | 53565 (1.18%) | 4482864 (98.82%) | 58800511 (85.37%) | 436127 (0.74%) | 58364384 (99.26%) |
| ST6d_3 | 70687076 | 6821218 (9.65%) | 96623 (1.42%) | 6724595 (98.58%) | 57183041 (80.90%) | 601745 (1.05%) | 56581296 (98.95%) |

ST and SC represent the inoculated and non-inoculated samples, respectively. 3d, 3 days post inoculation (dpi); 6d, 6 dpi. Each group contained three biological replicates.
